# Multiple Self-Healing Effects of Water-Absorbing Microcapsules in Cementitious Materials

**DOI:** 10.3390/polym15020428

**Published:** 2023-01-13

**Authors:** Qianjin Mao, Jiayi Chen, Wenwen Wu, Runfeng Li, Shuqing Shi, Ziming Wang, Suping Cui

**Affiliations:** 1 Faculty of Materials and Manufacturing, Key Laboratory of Advanced Functional Materials, Ministry of Education of China, Beijing University of Technology, Beijing 100124, China; 2 State Key Laboratory of Solid Waste Reuse for Building Materials, Beijing Building Materials Academy of Science Research, Beijing 100041, China

**Keywords:** self-healing, microcapsule, water absorbing, cementitious material, environmental adaptability

## Abstract

Concrete cracking has a negative impact on the durability of the structure. Pre-implanting microcapsules containing healing agents into the concrete are expected to induce the cracks to self-heal. However, the self-healing effect can potentially be influenced by several environmental conditions, thus limiting its applications. To address these challenges, we developed a new type of water-absorbing microcapsules, using calcium alginate hydrogel as the wall material and an adhesive epoxy polymer as the core material, to improve the self-healing adaptability in complex and changing environments. We explored the healing properties and mechanism of cementitious materials containing microcapsules under various environmental conditions. The experimental results showed that the water-absorbent microcapsules exhibit multiple self-healing effects under different external conditions: (1) in an anhydrous environment, fissures prompted the activation of microcapsules, and the epoxy polymer flowed out to seal the cracks. (2) When exposed to water, the microcapsules inflated to form a seal around the fissures. (3) The microcapsules facilitated the autogenous healing of cracks in the cementitious material when wet and dry conditions were alternated. The three self-healing mechanisms worked synergistically and contributed to the effective restoration of the impermeability and strength of concrete under different environments. Particularly, the recovery of compressive strength and impermeability exceeded 100% when the microcapsule content was 4% and the pre-pressure was 40% of f_max_.

## 1. Introduction

Concrete is prone to cracking, which significantly lowers its durability and has a significant impact on its production and application [1,2]. Every year, countries invest a significant amount of money and resources in concrete crack repair [3,4]. However, mending cracks in the later stages of work have a negative effect; 20% of the repaired concrete will collapse after five years and 55% will fail after ten years [5].

In recent years, it has become critical to develop an intelligent material capable of successfully detecting and autonomously repairing cracks to address concrete’s serious cracking problem. Carolyn Dry [6] proposed the concept of self-healing concrete for the first time in 1994. Self-healing in concrete can be classified into two categories: autogenous healing and autonomous healing [7]. Autogenous healing is the phenomenon of the crack healing intrinsically based on chemical reactions and the involvement of water. Due to the additional hydration of dry cement surrounding fractures and the dissolution of carbonation by Ca(OH)_2_, cracks become self-healed [8]. However, autogenous healing is constrained by the width of the crack and the number of dry cement particles, as well as by the environment and the duration of time required [9,10]. Generally, only cracks with a width of less than 50 µm can be entirely mended by autogenous healing, therefore the effect is less than optimal. As a result, some form of promotion or other self-healing strategy is required. Autonomous self-healing is the process of cracks automatically healing when particular compounds are added to cementitious materials. Self-healing materials mimic the mending process of damaged biological tissue. Special components are pre-incorporated within the matrix; when the matrix sustains cracks, the repair system buried within, releases the healing agent in response to force, heat, or chemical damage, hence plugging the crack in its initial stage and preventing it from spreading further. There are numerous known methods of self-healing concrete at the moment, including the addition of mineral admixtures [11,12,13], engineering cementitious composites [14,15,16], bacteria [17,18,19], microcapsules [20,21,22], superabsorbent polymers [23,24,25], shape memory alloys [26,27,28], vascular networks [29,30], and other methods [31].

Microcapsule technology involves the pre-implantation of microcapsules containing a healing agent into the solid matrix material. When fractures form, concentrated tension causes the microcapsules near the crack tip to rupture, allowing the healing agent to flow out. White [19] first examined microencapsulation techniques utilizing dicyclopentadiene as a wound healer and urea-formaldehyde as the shell material. Following that, it penetrates the matrix fracture by capillary action, solidifies, and fills the crack. This procedure has the effect of restoring material characteristics.

Numerous types of microcapsules have been developed as a result of the advancement of microcapsule technology. The commonly used core materials include sodium silicate microcapsules [21], isocyanate microcapsules [32], epoxy resin microcapsules [33,34], and other healing agents [35]. The commonly utilized wall materials include urea-formaldehyde resin [36,37], polyurethane [21], etc. [38]. Many factors affect the self-healing characteristic of cement. These include the content of the microcapsules in the cement, the particle size of the microcapsules, the degree of damage, and the healing time and conditions. When the conditions are suitable, the microcapsule shows a substantial effect on restoring the mechanical properties and impermeability of the concrete. According to Li [39] and Tittelboom [40], by adding microcapsules to concrete, the mechanical strength can be restored to between 130 and 140% of its original value following conventional curing. According to Feng [41], adding microcapsules to concrete can restore its impermeability; the recovery ratio can exceed 90% after 28 days of healing.

Although microcapsules have a self-healing effect in the laboratory, the external environment in practical applications is variable, and microcapsules must adapt to numerous environmental changes in order to exert a good self-healing effect. Most microcapsules, however, are employed in a single setting. For example, a few experiments were conducted under standard curing conditions [42], while others were conducted under water curing conditions [43]. Since the self-healing effect of many microcapsules is tested under a specific healing condition, the self-healing effect in other circumstances is unknown. Therefore, it is unclear how changes in the external environment will impact the self-healing ability of microcapsules. It is very important to construct microcapsules with high environmental tolerance.

Superabsorbent polymers (SAPs) are a type of cross-linked hydrogel consisting of water-soluble polymers that can absorb hundreds of times the weight of a water solution from the surrounding environment [44] and retain or release water according to the environment. When water enters a fracture, SAPs absorb it and expand, sealing the fissure and preventing the entry of further ions. Under dry conditions, SAPs can release water to the crack surface, where it reacts with unhydrated cement particles, promoting secondary hydration [45,46,47]. The products are CaCO_3_ and small amounts of Ca(OH)_2_ and CSH as well as CASH [48], which can contribute to the subsequent healing of cracks. Owing to these unique properties, SAPs can help self-healing of concrete cracks [49].

SAPs are used as a shell material to make self-healing microcapsules. These self-healing microcapsules can perform both autogenous and autonomous self-healing, hence accelerating and increasing their self-healing capabilities. Thus, they can be utilized in different conditions. With hydrogel calcium alginate as the wall material and epoxy resin as the core material, a new self-healing microcapsule system has been developed. The microcapsules’ structure, strength, and water-absorbing characteristics [50], as well as their effects on mortar shrinkage reduction and concrete workability [51], have been investigated. It has been established that water-absorbent microcapsules can improve the concrete’s resistance to cracking and have less influence on the workability of concrete.

Research on water-absorbing microcapsules needs to be conducted in detail. This article examines the self-healing mechanism of water-absorbing microcapsules. The self-healing capacity of the shell and core in diverse conditions has been examined, in addition to their role in restoring the material properties. The optimal microcapsule component capable of being utilized in various environments is also being explored.

## 2. Materials and Methods

### 2.1. Materials

Sodium alginate (SA) and epoxy resin (E-51) were used to create SAP microcapsules. Raw materials used for the experiments have been listed in Appendix A.

The cement and sand used in this paper were the same as the reported study [51] to ensure the consistency of the experiments.

### 2.2. Specimens

#### 2.2.1. Preparation of Microcapsules

Calcium alginate–epoxy microcapsules were synthesized using the sharp hole-solidification bath method. The precise preparation process and ingredients have been detailed in the previous study [51]. A total of 1.6 weight% of sodium alginate was added to deionized water and mixed for 1 h in a 60 °C thermostatic water bath. Then, sodium dodecyl phenyl sulfonate was added and mixed for 10 min. Next, specific weights of E-51 epoxy resin and diluent were added and the system was allowed to stir for 1 h. Thereafter, the mixture was dripped into a calcium chloride solution through an orifice mold. The mixture was then allowed to stand for 3 h after which solidification of the microcapsules takes place. The microcapsules were finally washed with anhydrous ethanol and dried at 60 °C.

A certain amount of dry microcapsules was weighed and the weight was referred to as *M*_0_. The microcapsules were thoroughly ground and immersed in acetone to completely remove the epoxy resin. The obtained capsule shell was filtered, dried, and weighed and this weight was recorded as *M*_1_. The core content of the microcapsule is calculated by Equation (1). The measurement was taken thrice for each sample and the average value was recorded.

(1) $$Core\;Content\;=\;\frac{M_{1}-M_{0}}{M_{0}}$$

A certain amount of dry microcapsules was weighed and the weight was recorded as *M*_0_. These microcapsules were put into a tea bag, which was then immersed in deionized water. The tea bag was taken out at intervals and weighed after draining the water completely until the weight remained unchanged. This weight was recorded as *M*_2_. The water absorption rate of the microcapsules is calculated by Equation (2). The measurement was repeated for three separate samples and the average value was recorded.

(2) $$Water\;Absorption\;Rate\;=\;\frac{M_{2}-M_{0}}{M_{0}}$$

The average diameters of microcapsules were found to be 0.7 ± 0.05 mm. The ratio of shell–core raw materials and the content of the core are shown in Table 1.

#### 2.2.2. Preparation of Paste Specimens

To prepare cement paste specimens, the cement to water ratio was 1:0.5, with microcapsules account for 3.5% of the cement weight and epoxy-curing agent accounting for 15% of the microcapsules weight. The paste of the control groups did not contain microcapsules but the other conditions were the same. The size of the cast prismatic mold was 40 mm × 40 mm × 160 mm. After standard curing (20 °C, RH = 95 ± 5%) for 24 ± 2 h, the mold was removed and the paste continued to dry until curing (20 °C, RH = 55 ± 5%) for 28 days. The prepared specimens were used to study the self-healing of cracks in cementitious materials.

#### 2.2.3. Preparation of Mortar Specimens

To prepare mortar samples, the cement to water was 1:0.5, the cement to sand was 1:3, the content of microcapsules was 0–5% by weight of the cement, and the amount of epoxy-curing agent was 15% by weight of the microcapsules. Mortars were poured into prismatic molds of size 40 mm × 40 mm × 160 mm, and three specimens were made for each group. After standard curing (20 °C, RH = 95 ± 5%) for 24 ± 2 h, the mold was removed and curing to a specific age. The prepared samples were used for compressive strength and self-healing tests.

The mortars were cast into truncated cone molds with a top diameter of 70 mm, a lower diameter of 80 mm, and a height of 30 mm. Six samples were made for each group. After standard curing (20 °C, RH = 95 ± 5%) for 24 ± 2 h, the mold was removed and the mortars were cured at 20 °C, RH = 55 ± 5% to a specific age. The prepared samples were used for impermeability and self-healing tests.

### 2.3. Testing Methods

#### 2.3.1. Optical Microscopy (OM)

After the paste samples reached a specific age of curing, the three-point bending method was utilized to manufacture micro-cracks. Using a stereomicroscope (OLYMPUS BX51, MicroPublisher 3.3 RTV, Tokyo, Japan), the surface cracks of the cement matrix were examined.

#### 2.3.2. Scanning Electron Microscopy (SEM)

After curing the paste samples to a particular age, they were shattered into 2–3 mm test pieces for Scanning Electron Microscopy (SEM) (Hitachi, SU9000, Tokyo, Japan) and EDS elemental analyses. The samples were dried in a 60 °C vacuum oven for three days and then sprayed with gold prior to SEM testing.

#### 2.3.3. Three-Dimensional X-Ray Computed Tomography (3D-XCT)

The cement paste specimens containing microcapsules were prepared with a water-to-cement ratio of 0.5. The specimen was produced with dimensions of 20 mm × 20 mm × 40 mm. These specimens were further cured at 55 ± 5% RH and 20 ± 2 °C. ZEISS Xradia 520 Versa (Carl Zeiss Microscopy, LLC, Peabody, MA, USA) was then utilized to visualize microcapsules in the hardened paste. The changes in the volumes of the microcapsules were further examined with different hydration durations.

#### 2.3.4. Mechanical Testing

The mortar was cured in water for 7 and 28 days. After a certain number of days, the compressive strength and flexural strength of the mortar were evaluated according to the national standards of the People’s Republic of China GB/T 17671-1999. Three specimens in each group were taken for a flexural strength test to break the specimen into two columns. Compressive strength testing of specimens was conducted after flexural strength testing. Six specimens in each group were taken for the mortar compressive strength tests. The compressive surface consisted of the two sides of the specimen during the forming and the process, with dimensions of 40 × 40 mm^2^.

To acquire pre-damaged mortar samples, various loading pressures were applied to the mortar. The pre-damaged mortar samples were cured under various settings. Some were placed in the curing room (20 ± 5 °C, 55 ± 5% RH) for self-healing for 14 days. The remaining items were cured for a total of 7 dry–wet cycles for 14 days; meaning that the samples were cured in a curing room (20 °C, 55 ± 5% RH) for 24 h and subsequently cured in water for 24 h. The specimens were finally reloaded. During the entire loading process, the load was done with the uniform speed of 2400 N/s ± 200 N/s until the specimen was damaged.

#### 2.3.5. Water Permeability Testing

The water permeability of the mortar was further tested according to the industrial standards of the People’s Republic of China, JGJ/T 70-2009. The specimens were first sealed with melted paraffin wax. The mortar penetrometer was then mounted for permeability testing. The initial test pressure was 0.2 MPa, which lasted for two hours before being increased to 0.3 MPa. After reaching 0.3 MPa, the pressure rose by 0.1 MPa each hour. When 3 of 6 samples were infiltrated, the test would end. The impermeability coefficient of the mortar test specimens was calculated using Equation (3).

(3) $$I=\sum P_{i}\cdot T_{i}$$

where *I* is the impermeability value of mortar, MPa·h; *P_i_* is the water pressure at each pressure stage, MPa; and *T_i_* is the duration of the corresponding pressure stage, h.

## 3. Results and Discussion

### 3.1. Characterization of the Microcapsule and Its Morphology in Cement Matrix

**Figure 1:**
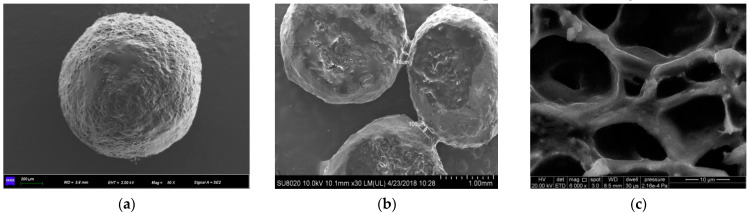
Characterization of microcapsules: (**a**) microcapsule morphology; (**b**) cross-section view of microcapsules; (**c**) interior of microcapsule with epoxy removed.

The characterization details of the microcapsule are shown in Figure 1.

**Figure 2:**
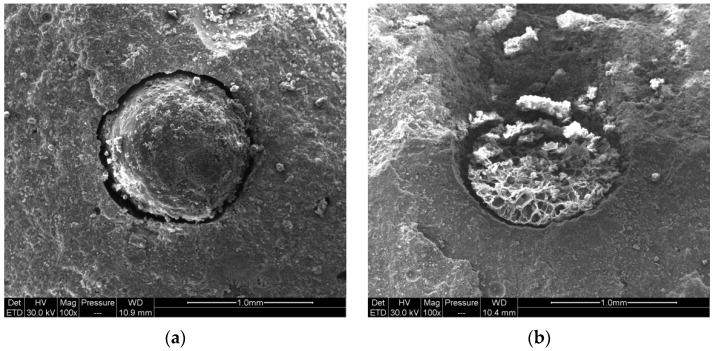
State of microcapsules in cement matrix: (**a**) complete microcapsule; (**b**) broken microcapsule.

Figure 1a shows that the microcapsule has a spherical and rough surface at the microscopic level. From Figure 1b, it can be seen that there is adhesion between the two neighboring half capsules, caused by the epoxy outflow from the core. Figure 1c shows that there is a three-dimensional network structure inside the microcapsules, and the core material exists between the calcium alginate networks, filling the pores. The calcium alginate/epoxy resin microcapsules with a networked scaffold structure can not only reduce the water absorption and swelling capacity of calcium alginate gel but also have more stable mechanical strength than the microcapsules with a general core–shell structure. The state of the microcapsules in the cement matrix is shown in Figure 2.

Figure 2a shows the embedded microcapsule undergoing agitation in the cement matrix. Figure 2b shows a broken microcapsule in the cement matrix. The internal network structure of the microcapsule can be observed in Figure 2b due to the outflow of epoxy from the ruptured microcapsule. It can be seen in Figure 2 that there are small pores at the interface between the microcapsules and cement. This is because the microcapsule wall material, calcium alginate, contains a large amount of hydroxyl (-OH), which absorbs water and expanded during the mixing process. With the decrease in internal humidity during cement hydration, calcium alginate gradually releases water and shrinks.

### 3.2. Multiple Self-Healing Mechanisms of Water-Absorbing Microcapsules

#### 3.2.1. Microcapsules Core Healing Agent Bonded Cracks in a Dry Environment

**Figure 3:**
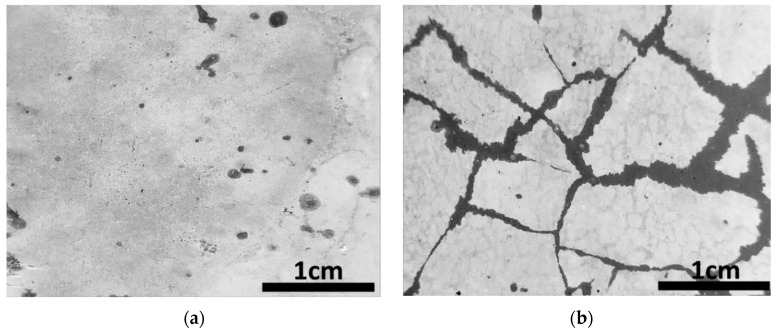
The surface of the sample containing microcapsules (**a**) before and (**b**) after loading.

After 28 days of curing, a three-point bending test was utilized to create microcracks in a cement paste sample and monitor the cracks’ self-healing process. Figure 3 shows a surface comparison of the sample before and after loading, with Figure 3b obtained 15 min after the loading was completed.

The dark regions in Figure 3b were epoxy resin-filled cracks. It was shown that when the sample broke, the calcium alginate microcapsules ruptured, releasing the adhesive, and the glue swiftly penetrated along the microcracks.

**Figure 4:**
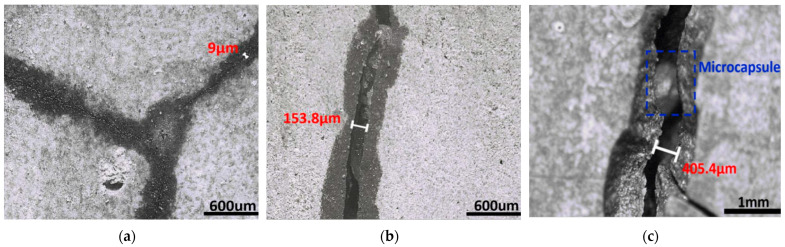
Filling effect of the epoxy healing agent in microcapsules on cracks with different widths. (**a**) The photograph showed that the cracks with a width of 0–20 μm were filled with epoxy resin; (**b**) cracks below 200 μm in width were partially filled by epoxy resin; (**c**) cracks with a width of about 400 μm failed to be filled by epoxy resin.

A stereo microscope (OM) was used to observe the process of the epoxy filling cracks of different widths. Figure 2 shows the filling effect of epoxy resin on cracks with widths of 0–20 μm, 20–200 μm, and greater than 200 μm. Figure 4 shows the state of a microcapsule in the cement.

Figure 4a clearly shows that epoxy resin is produced in the fractures with a width of 0–20 µm, with a large diffusion depth and the crack region is filled. The microcrack serves as a capillary when the crack width is small. It demonstrates that capillary pressure is the primary driving factor of epoxy resin entering into microcracks and that capillary pressure is inversely related to the capillary radius [52,53]. The narrower the width of the crack, the greater the capillary pressure and the greater the depth of liquid intake. As a result, small cracks of 0–20 µm width can generate enough capillary pressure to allow the epoxy resin to flow relatively long distances and therefore fill the entire microcrack.

Figure 4b shows that for cracks of 20–200 µm width, microcapsules can still rupture upon cracking, releasing adhesive to mend the crack, but the epoxy resin cannot fill the entire crack; only the segment close to the microcapsule can be filled. This is due to the limited content of the microcapsule core, which is not sufficient to fill the entire crack area for wider cracks. Furthermore, when the fracture grows wider, the capillary phenomenon weakens, resulting in the core material being unable to enter farther along the fissure and only sealing some cracks.

The microcrack with a width of 400 µm continues through the microcapsule, and the morphology of the microcapsule remains intact. The outflow of the epoxy core can still be detected, but it cannot play the role of crack sealing, as illustrated in Figure 4c. It demonstrates that the healing effect of a microcapsule adhesive bond fracture is inadequate for wide-width cracks due to the inadequacy of the total amount of healing agent. Wide cracks will rely mostly on the water absorption-release and swelling self-sealing of the microcapsule to promote self-healing.

**Figure 5:**
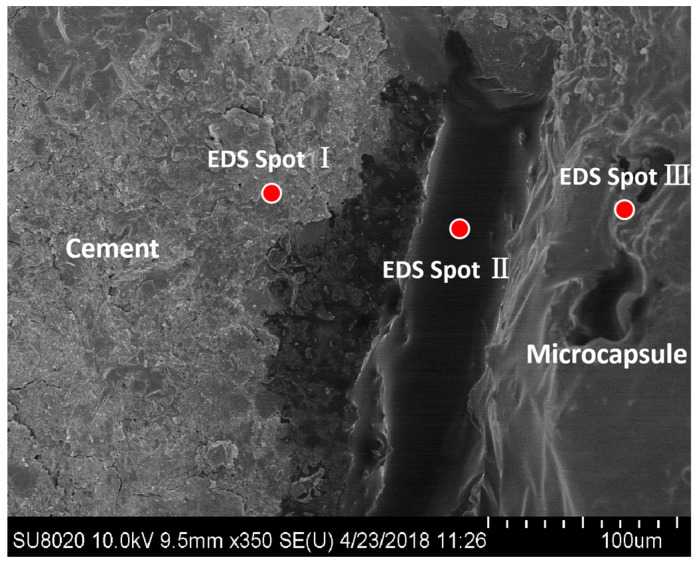
SEM image of the crack in the paste sample containing microcapsules [50].

Using a scanning electron microscope and EDS energy spectrum, the area where the epoxy flowed out to fill the fracture was examined; the same results were also observed in our earlier study [49]. The SEM micrographs of the microcapsules’ break area are shown in Figure 5. In the cement matrix, fracture, and microcapsule regions, points I, II, and III, respectively, were selected. These findings are shown in Table 2.

Table 2 shows that point I is the cement matrix and the primary elements present are Ca, Si, O, C, and Al. Point III is the microencapsulated calcium alginate. The content of element C at crack zone II is much higher than that at points I and III. It can thus be deduced that epoxy resin is present. The element N in this area is derived from tetramethylene pentamine, which is the epoxy-curing agent. It demonstrates the curing response of the epoxy resin and curing agent. According to the analysis presented above, when the microcapsules ruptured, epoxy resin entered the fracture zone and was able to interact and react with the curing agent dispersed throughout the cement matrix, sealing the crack.

#### 3.2.2. Microcapsule Swelling to Seal Cracks in a Water Environment

**Figure 6:**
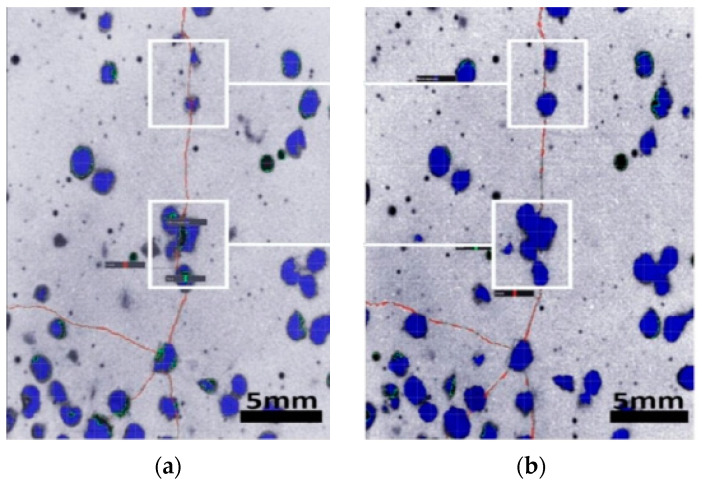
XCT image of microcapsules and cracks in cement paste specimen [50]; (**a**) cracks through microcapsules; (**b**) microcapsule swelling sealed the cracks.

After curing for 28 days, specimens containing microcapsules were squeezed until the fractures developed and then immersed in water for 7 days. Using XCT tomography, the morphology of the microcapsules in the cement matrix was examined. Microcapsules and cracks can be recognized and colored because materials with varied densities have varying grey values when exposed to X-rays. The microcapsules were dyed blue, and the cracks were colored red, as shown in Figure 6.

As illustrated in Figure 5, cracks ran throughout the specimen, implying that water might enter directly through cracks. After 7 days of soaking, the volume change of the microcapsules surrounding the cracks gradually decreased in the direction in which the fissures extended from the outside to the center. The microcapsules in white boxes (Figure 4) had significantly expanded in size. These observations demonstrated that the microcapsules expanded after absorbing water, thereby preventing the passage of further water into the matrix. These experimental findings demonstrated that the water-absorbent microcapsules are capable of self-sealing.

#### 3.2.3. Microcapsules Promoting Autogenous Healing in Dry-Wet Cycles Environment

**Figure 7:**
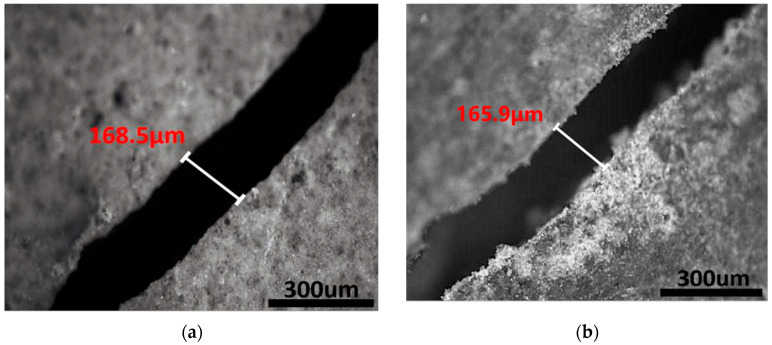
The crack in the reference specimen before and after healing for 28 days: (**a**) before the dry and wet cycle; (**b**) after the dry and wet cycle.

**Figure 8:**
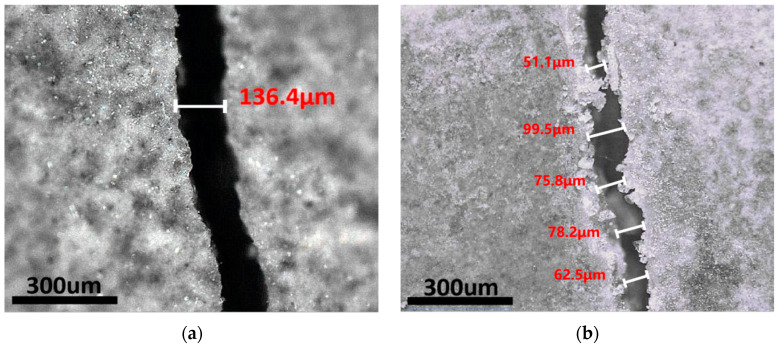
The crack in the sample containing microcapsules before and after healing for 28 days: (**a**) before the dry and wet cycle; (**b**) after the dry and wet cycle.

The self-healing effect of the microcapsule shells is investigated in this section. Thin steel sheets were inserted into the fresh paste and then pulled out before the paste solidified, thus creating gaps. After curing for 28 days, the specimens were treated for another 28 days under dry–wet cycle conditions. Figure 7 depicts a comparison of cracks before and after the healing of reference specimens without microcapsules, while Figure 8 depicts a comparison of cracks before and after the healing using microcapsules.

Figure 7 shows that the fracture width remained nearly unchanged after the dry and wet cycles. Figure 7 shows that after 28 days of dry and wet cycle maintenance, the fracture was filled with fresh material, narrowing the crack width but failing to seal the crack completely. According to the literature, autogenous healing can only cure cracks less than 50 µm in length [8,54,55], and its efficiency is substantially lower than that of autonomous self-healing. These experimental results demonstrated that the microcapsules significantly promoted autogenous healing.

**Figure 9:**
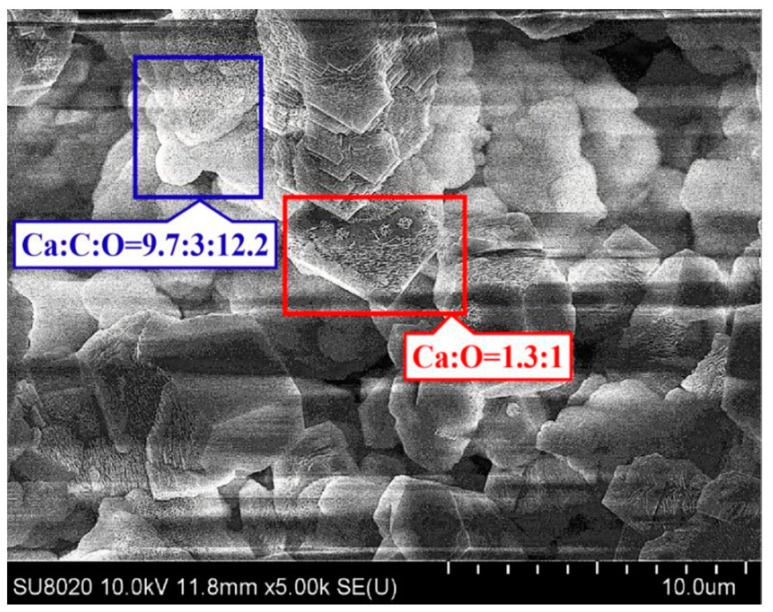
SEM image of the crack area after autogenous healing.

SEM and energy spectrum analyses were used to examine the products in the fracture area, which is shown in Figure 9.

Figure 9 shows that the sediments in the fractures were primarily hexagonal calcium hydroxide and spherical calcium carbonate crystals, with fewer cement hydration products, which was consistent with the conclusion of the literature [23,56]. This is primarily because calcium alginate microcapsules absorb water in a moist environment and slowly release it during drying, causing precipitates to accumulate in the cracks. Ca(OH)_2_ in the cement matrix diffuses and dissolves in the water of the crack when the cement is constantly in the presence of water, and precipitates on the crack surface when the concentration reaches saturation. Furthermore, the Ca(OH)_2_ in the water interacts with CO_2_ to generate CaCO_3_, which is deposited on the crack surface. The Ca(OH)_2_ and CaCO_3_ microcrystals filled in the cracks, preventing water entry and restoring matrix impermeability.

### 3.3. Compressive Strength Healing Effect

#### 3.3.1. The Influence of Loading Pressure on the Compressive Strength Healing Effect

**Figure 10:**
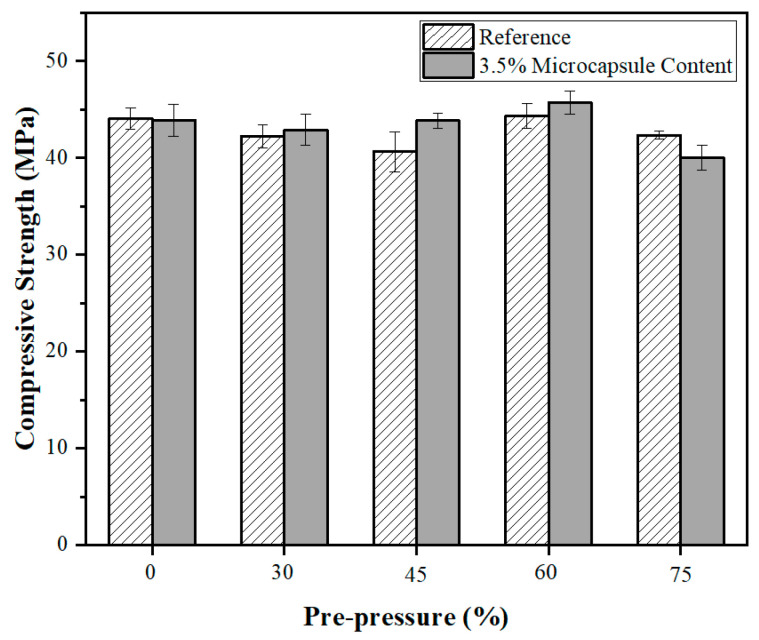
Compressive strength of mortar samples with different pre-pressure.

Microcapsules at a concentration of 3.5% of cement grade were added to the cement mortar. After standard curing for 21 days, specimens were preloaded with 0%, 30%, 45%, 60%, and 75% of f_max_ (f_max_ was the strength of the crushed specimens). Figure 10 depicts the evaluation of compressive strength 7 days after standard curing following 2 min of loading pressure. The control denoted an absence of microcapsules in the sample.

When the preloading was 45% of f_max_, as depicted in Figure 10, the compressive strength of the microcapsule mortar specimens after healing was virtually equal to that of the unloaded specimen. This proved that the microcapsules restored the compressive strength of the cement-based composites. The rupture of the microcapsules was hence sensitive to preload. When the preloading force reached 45–60% of f_max_, the majority of the microcapsules were ruptured, and the healing agent flowed out to glue the fractures, restoring the mechanical properties of the matrix. When the preloading force was increased to 75% of f_max_, the compressive strength of the samples containing microcapsules was lower than that of the reference samples and its initial strength. Under the influence of 75% pre-pressure, a significant number of cracks formed in the cement matrix. This is consistent with the findings of other researchers [57]. As a result, the microcapsules’ self-healing ability was insufficient to fix the cracks in the cement matrix. In this approach, the presence of microcapsules is analogous to introducing faults into the cement matrix, resulting in samples with less strength than the control without microcapsules.

When the pre-pressure is less than 45% of f_max_, the strength of the reference cement decreased with the increase in pre-pressure. This is because the pre-pressure brings internal damage to the matrix. Meanwhile, the strength of cement with microcapsules increased due to the self-healing effect. When the pre-pressure is between 60% and 75% of f_max_, the matrix density increased, increasing the secondary strength of the reference specimen. As the pre-pressure grew from a low value, the secondary compressive strength of the reference specimen decreased.

#### 3.3.2. The Influence of Microcapsule Content on Compressive Strength Healing Effect

**Figure 11:**
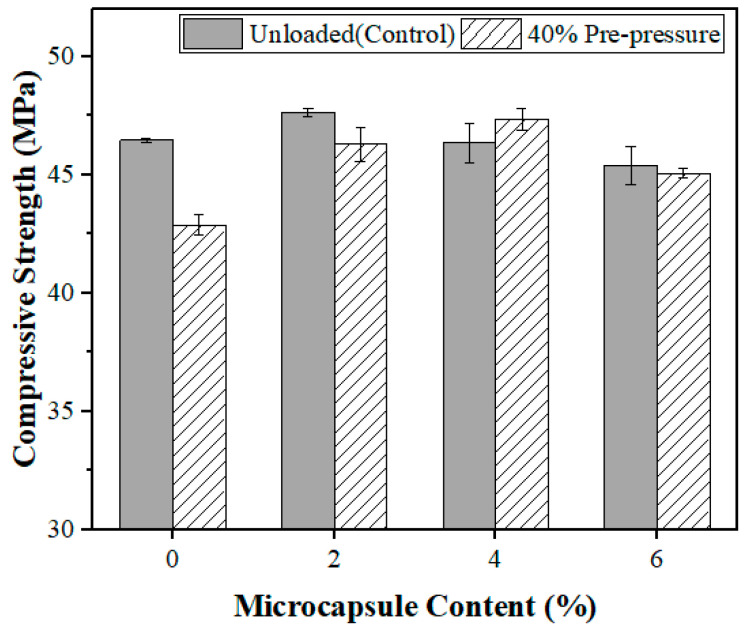
Compressive strength of mortar samples with various microcapsule contents.

According to the results obtained in Section 3.3.1, the microcapsules have a certain self-healing effect when the pre-pressure is less than 60%. Due to the interference of certain matrix compaction under 60% pre-pressure, 40% pre-pressure was selected as the optimized value to explore the effect of microcapsule content on the self-healing effect under the same pre-pressure condition. The microcapsule-B contents in the mortar specimens were 0, 2, 4, and 6% of the cement weight. On day 28 of standard curing, the specimens were preloaded up to 40% of their maximum load-carrying capacities for 2 min. Their compressive strength was tested after 14 days of dry curing (20 °C, RH = 55 ± 5%) for the samples with and without preload, as shown in Figure 11.

Figure 11 demonstrates that the compressive strength of the reference specimens decreased by approximately 7.70% when subjected to 40% f_max_ preload compared to the unloaded specimens. With a 2% microcapsule addition, the compressive strength of the self-healing mortar specimens was 2.86% lower in comparison to that of the unloaded specimens. When the addition of microcapsules was between 4% and 6%, the compressive strength of the self-healing mortar specimens exceeded that of the uncompressed specimens. Under the parameters of the experiment, the compressive strength could be fully restored at an addition of microcapsules equivalent to 4–6%. The self-healing effect is insufficient when the microcapsule content is low, and when microcapsule content is excessively high, the excess microcapsules are equivalent to defects. According to our experiment, the optimal content of microcapsules in the cement should be 4%. In addition, microcapsules of calcium alginate are found to have the best self-healing effect on cementitious materials. The self-healing effect is similar to the results reported earlier [58], although the test methods are not entirely the same.

#### 3.3.3. Self-Healing Effect in Drying Curing

**Figure 12:**
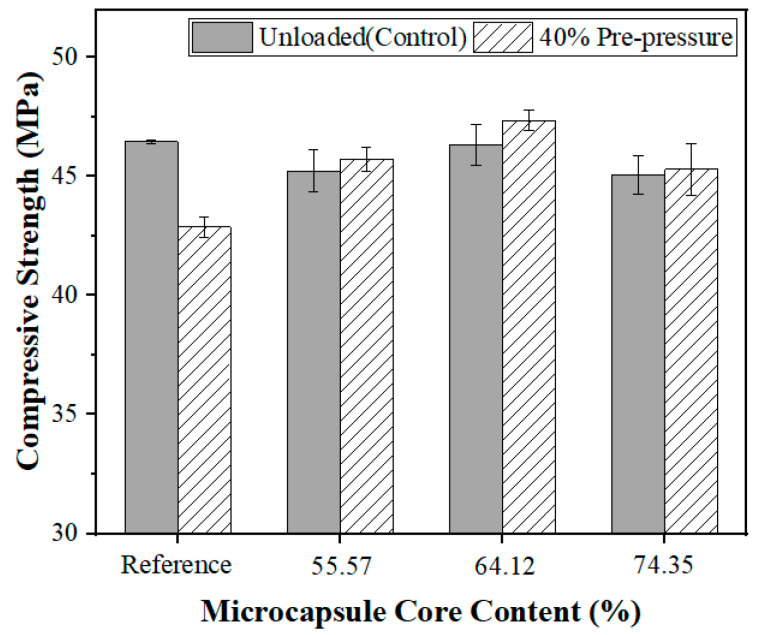
Self-healing effect under dry curing conditions.

The shell-to-core ratio of microcapsules also influences the self-healing effect. Microcapsules-A, B, and C with 4% of cement weight in the mortar were employed in the test. The self-healing effect of compressive strength under dry curing conditions is shown in Figure 12.

As illustrated in Figure 12, microcapsules with a higher core content exhibited a better self-healing effect under dry curing conditions. These experimental results are consistent with other literature reports that document the healing rate on compressive strength of epoxy resin microcapsules after 14 d drying curing ranging between 70% and 110% [41,55]. The self-healing agent bonded the cracks, thereby contributing to the self-healing effect (see Section 3.2.1). When the microcapsules are ruptured by cracks, the healing agent flows out and reacts with the epoxy-curing agent to bind the cracks.

It is worth noting that the self-healing effect of the microcapsules with 74.35% core content was lower than that of the microcapsules with 64.12%. A plausible explanation for this observation is that too high of a core content leads to epoxy deposition on the surface of microcapsules (a poor flowability of microcapsules was observed in the experiments), which in turn reduces the deposition of cement hydration products on their surface, thus making the interfacial bonding weaker and reducing the self-healing effect.

#### 3.3.4. Self-Healing Effect in Dry–Wet Cycles Curing

**Figure 13:**
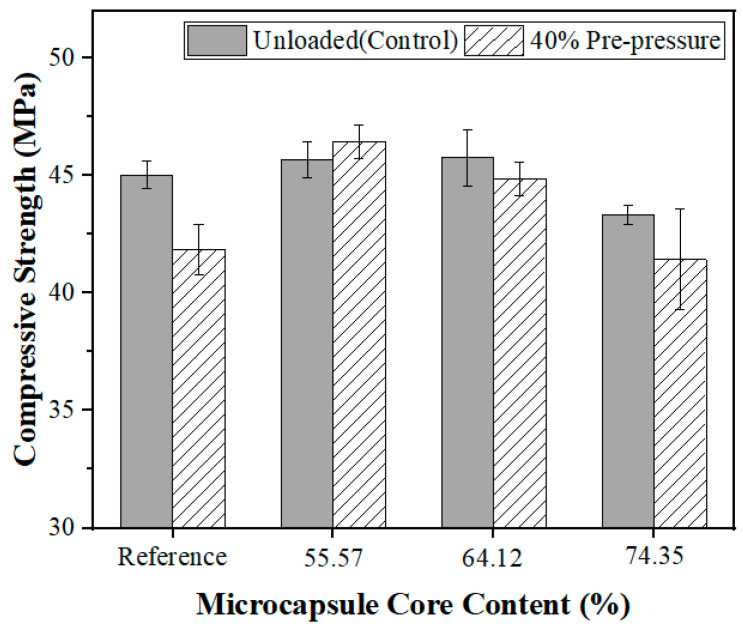
Compressive strength in dry–wet cycles healing situation.

Microcapsules demonstrate different self-healing effects in different environments. Therefore, the self-healing effect of the microcapsules under changing wet and dry conditions was investigated. A dry–wet cycle comprising 24 h of drying and 24 h of immersion in water was established. Microcapsules-A, B, and C with 4% of cement weight in the mortar were used in the test. The self-healing effects corresponding to the recovery of compressive strength upon exposure to dry–wet cycles are shown in Figure 13.

Figure 13 demonstrated that the self-healing effect was enhanced when microcapsules were added to the mortar samples, indicating that the microcapsules had a self-healing effect under dry–wet cycle conditions. The observed self-healing effect is better than using SAPs alone [59]. This implies that both the healing agent core and the water-absorbing polymer wall contribute to self-healing under conditions involving exposure to dry–wet cycles. As shown in Figure 12b, when the core content of the microcapsules increased from 55.57% to 74.35% under the dry–wet cycles curing conditions, the self-healing efficacy of microcapsules was reduced. It suggested that the microcapsule wall had a bigger impact on the self-healing effect than the core under these experimental conditions. It may be concluded from this result that the established cyclic wet–dry conditions, which prevented the inside of the microcapsules from drying completely, also hindered the curing of the epoxy, thus weakening its self-healing effect. In contrast, the function of the walls to promote autogenous healing was most effective when exposed to cyclic wet–dry conditions.

### 3.4. Water Permeability Healing Effect

**Figure 14:**
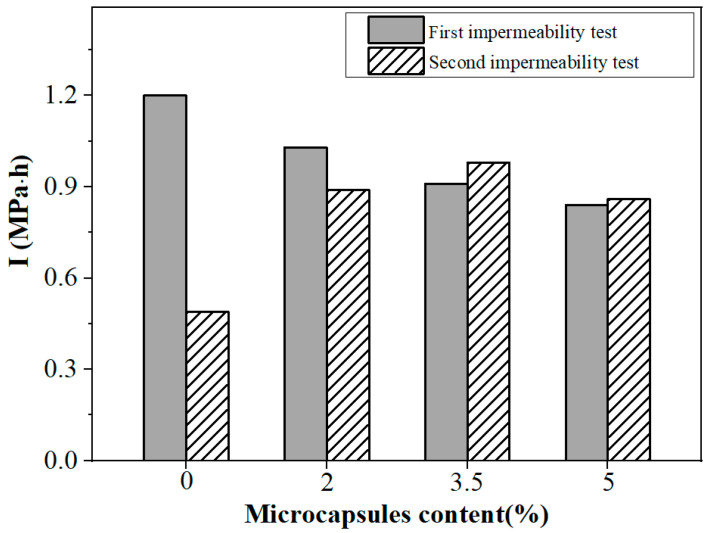
Impermeability coefficient of mortar specimens with different microcapsule content.

The self-healing of the damage caused by water pressure to mortars containing microcapsules has been investigated below. The microcapsules with a core content of 64.12% were added to mortar specimens at 0%, 2%, 3.5%, and 5% of cement mass. Following 28 days of dry curing, the first impermeability test was conducted. After the first impermeability test, the sample used in the first test was allowed to cure for 14 days under cyclic dry and wet conditions to heal the water passage caused by water pressure in the first impermeability test. Subsequently, the secondary impermeability test was performed. The two permeability tests, depicted in Figure 14, determined the impervious coefficient values for mortar samples with various microcapsule contents.

The I-value of the first impermeability test primarily reflected the mortar specimen’s intrinsic impermeability. As shown in Figure 14, the impermeability I-value of microcapsule cement specimens decreased with increasing microcapsule content as compared to the control group. This may be attributed to the presence of microvoids between the microcapsules and the cement matrix, thus the microcapsules did not have sufficient time to swell during the impermeability test, resulting in a weakened impermeability of the matrix. The I-value for the second impermeability test corresponds to the impermeability of mortar specimens following water pressure damage and 14 days of dry–wet cycles, showing the mortar specimens’ capacity for self-healing. The second impermeability of control specimens had lower I-values than the first impermeability, with the net decrease equivalent to 59%. I-values decreased due to the formation of water-infiltrated pathways during the first test, which continued to form during the second test. For specimens containing 3.5–5% microcapsules, the second impermeability was significantly higher than the control group, even exceeding its first I-value. The primary reason for this observation is that the synergistic effect of both the core outflow blocking the water channels and the capsule walls promoting autogenous healing. Another reason is the self-sealing effect of the microcapsules, which absorb water and swell, blocking the water channels. As a result, the I-values of the second impermeability test of specimens containing microcapsules was drastically enhanced.

**Figure 15:**
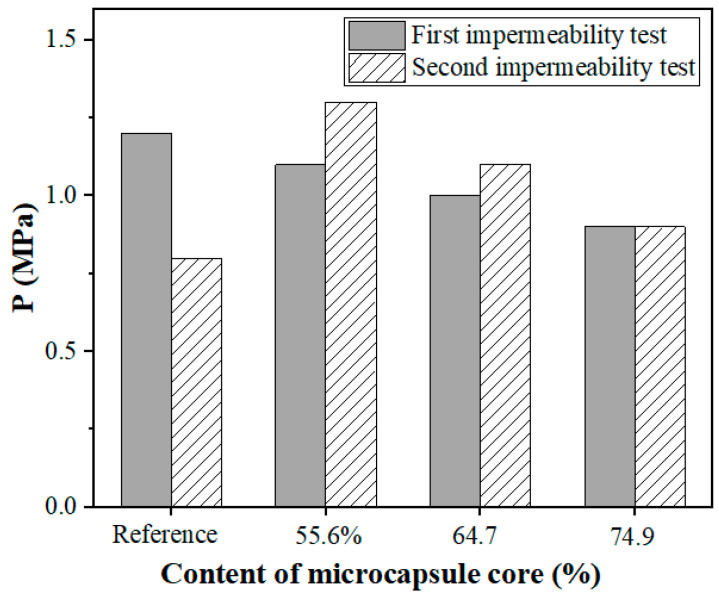
Impermeability coefficient of mortar specimens with different microcapsule core content.

Figure 15 shows the results of the water permeation tests for microcapsule samples with different core contents. Microcapsules-A, B, and C with 4% of cement weight in the mortar were used in the test.

As shown in Figure 15, the microcapsules with a core content of 55.6% had the best self-healing effect, and the self-healing effect decreased with the increase in the microcapsule core content, which was consistent with the results in Figure 13. This outcome implies that in addition to the dry and wet cycling conditions favoring the self-healing effect of the wall, the overall self-healing effect of the impermeability is further enhanced by the self-sealing effect brought by the swelling of the microcapsules. Thus, the water-absorbing microcapsules exhibited excellent impermeability recovery.

## 4. Conclusions

Based on the results of this experimental investigation, the following conclusions are drawn:

1. Three types of self-healing cooperation for water-absorbing microcapsules were observed. Different roles are played by the SAP microcapsule depending on the external environment. First, in a drying environment, cracks can trigger microcapsules to break, the epoxy resin bonds the microcracks, resulting in quick healing of fine cracks (less than 200 μm). Second, during exposure to changing dry–wet environmental conditions, water-absorbing microcapsules can promote the autogenous healing of cementitious materials. Third, in a wet environment, unbroken microcapsules can absorb water by swelling and plugging fractures, preventing additional water infiltration, and sealing macro cracks (more than 200 μm). Thus, regardless of the environmental conditions, water-absorbing microcapsules can promote the self-healing of cementitious materials.

2. These triple actions will contribute separately. When the microcracks are produced and expanded, the microcapsule distributed along the cracks ruptures and binds the cracks quickly. The main contribution comes from the curing of the epoxy resin and the bonding effect on the crack surface. Epoxy resin flows out into cracks and matrix pores to improve the strength and impermeability of the cement matrix. The initial healing occurs, which is also the primary healing mechanism of calcium alginate microcapsules. When the crack expands around the microcapsule, the microcapsule acts as a second healing mechanism by absorbing water, expanding and sealing the crack, promoting the third healing mechanism, and mineral crystallization precipitation to fill the crack. Second, the synergistic effect of the triple healing process allows for numerous healings of the same damaged site. The three restoration mechanisms work together to restore the impermeability and mechanical strength of the cement paste.

3. Utilizing absorbent microcapsules effectively restores the impermeability and compressive strength of cementitious materials. The compressive strength recovery rate exceeds 100% and the impermeability recovery rate exceeds 100% when the microcapsule content is 4% and the pre-pressure is 40% of f_max_.

## Figures and Tables

**Table 1:** The shell–core raw material ratios and core contents.

	Shell–Core Ratio	Core Content	Water Absorbing Rate
A	1:5.5	55.57%	39.86%
B	1:7.5	64.12%	30.12%
C	1:9.0	74.35%	24.01%

**Table 2:** Element composition of different areas in the crack (mass fraction/%) [50].

Point	Element Composition						
	C	O	Ca	Si	Al	Cl	N
I	3.25	31.98	42.15	11.73	2.05	0.00	0.05
II	59.90	24.15	9.04	0.00	0.00	2.20	5.11
III	29.12	19.69	28.08	0.00	0.00	22.58	0.01

## Appendix A

**Table A1:** Raw Materials Used for the Experiments.

Drug Name	Application	Pure Degree	Manufacturer
Sodium alginate	Preparation of Microcapsules	99.9%	Shanghai McLean Biochemical Technology Co., Ltd. (Shanghai, China)
Epoxy resin (E-51)	Preparation of Microcapsules	99.8%	Sinopec Baling Petrochemical Co., Ltd. (Hunan, China)
Cyclooxygen dilutions	Preparation of Microcapsules	-	Shanghai Aotun Huagong Technology Co., Ltd. (Shanghai, China)
Sodium dodecyl phenyl sulfonate	Preparation of Microcapsules	AR	Tianjin Forchen Chemical Reagent Factory (Tianjin, China)
Anhydrous calcium chloride (CaCl2)	Preparation of Microcapsules	AR	Beijing Tongguang Fine Chemical Co., Ltd. (Beijing, China)
Absolute ethyl alcohol	Washing of Microcapsules	AR	Beijing Tongguang Fine Chemical Co., Ltd. (Beijing, China)
Portland cement	Preparation of Mortarand Paste	P∙I 42.5	Qufu Zhonglian Cement Co., Ltd. (Shandong, China)
ISO standard sand	Preparation of Mortar	—	Xiamen Europe Standard Sand Co., Ltd. (Xiamen, China)
Tetraethylenepentamine	Preparation of Mortar	AR	Shanghai McLean Biochemical Technology Co., Ltd. (Shanghai, China)
